# Genome annotation of *Microbacterium* phage Statler containing a putative anti-CBASS gene (Acb2)

**DOI:** 10.1128/mra.00735-25

**Published:** 2026-06-01

**Authors:** Daniel Puentes Navarro, Sara Sadeghi, Jack F. Shurley, Anna S. Grinath, Michael A. Thomas

**Affiliations:** 1 Department of Biological Sciences, Idaho State University 6640, Pocatello, Idaho, USA; University of Pittsburgh School of Medicine, Pittsburgh, Pennsylvania, USA

**Keywords:** bacteriophages, *Microbacterium*, cyclic oligonucleotide sequestration protein Acb2, CBASS, SEA-PHAGES, INBRE

## Abstract

Statler, a *Microbacterium foliorum* bacteriophage isolated from grass thatch, belongs to Actinobacteriophage cluster EG with siphovirus morphology. Statler’s genome contains 108 genes and 62,248 bp of DNA with 66.9% guanine-cytosine (GC) content. The genome encodes a putative anti-CBASS gene, Acb2, predicted to inhibit the bacterial cyclic-oligonucleotide-based anti-phage signaling system (CBASS).

## ANNOUNCEMENT

Isolating and annotating new bacteriophages are fundamental for advancing phage research, as the resulting knowledge can be applied to medicine, agriculture, and biotechnology (1, 2). Here, we describe bacteriophage Statler, a lytic phage with siphovirus morphology discovered in a grass thatch outside Idaho State University’s Plant Sciences building (42.86734°N, 112.42904°W). Following Howard Hughes Medical Institute (HHMI) Science Education Alliance-Phage Hunters Advancing Genomics and Evolutionary Science (SEA-PHAGES) protocols (3–5), the sample was rinsed with 30 mL peptone yeast calcium agar (PYCa) liquid media, centrifuged at 2,000*g* for 10 min, and filtered with a 0.22 µm filter. The filtrate (~0.5 mL) was inoculated with 0.5 mL of *Microbacterium foliorum* NRRL B-24224 (6), then incubated and gently shaken for 48 h at 30°C. A 500 µL subsample was filtered and plated using a double agar overlay plaque assay with *M. foliorum* on PYCa agar, then incubated for 48 h at 20°C. After the plaques were identified, they were purified with three rounds of plating.

Using Promega Wizard DNA Cleanup Kit (A7280), Statler’s DNA was extracted from a high-titer lysate (>10^10^ PFU/mL) and prepared for sequencing using NEB Ultra II Library Kit with V3 reagents. Shotgun sequencing on an Illumina MiSeq generated 5,443,190 single-end 100 bp reads with 17,488-fold coverage. Raw sequencing reads were trimmed and assembled into a single contig using Newbler v2.9. Assembly completeness, accuracy, and phage termini were subsequently assessed with Consed v29; both programs run with default parameters (7, 8). The assembled genome is 62,248 bp with a 199 bp terminal repeat and a guanine-cytosine (GC) content of 66.9%. Statler was assigned to the cluster EG based on the similarity of its gene content to phages within the cluster in the Actinobacteriophage Database (9). The top BLASTn hit was FrankDeliGuy with a sequence similarity of 95%, another EG phage.

Statler’s genome was annotated with DNAMaster v5.23.2 (10) and refined with PECAAN (11). Phamerator v556 (12) was used for cluster assignment and comparison of gene synteny with previously annotated genomes. Gene prediction was supported by GeneMarkS and Glimmer (13, 14), while Starterator v1.2 provided start site comparisons with previously annotated EG phages (http://phages.wustl.edu/starterator/). HHpred V2.1,pdb, pfamA, National Center for Biotechnology Information (NCBI) CD, BLAST v2.15.0, PhagesDB, and NCBI databases and searches were used to identify putative gene functions (15, 16). DeepTMHMM v 1.0.42 predicted nine transmembrane proteins (17), and one tRNA was identified using Aragorn and tRNA-SE (18, 19). All software was run with default parameters.

Transmission electron micrographs revealed siphovirus morphology, with a capsid of ~55 nm and a tail length of ~160 nm (see representative TEM at PhagesDB). Plaques were small to medium-sized clearings (see example at PhagesDB).

**Fig 1:**
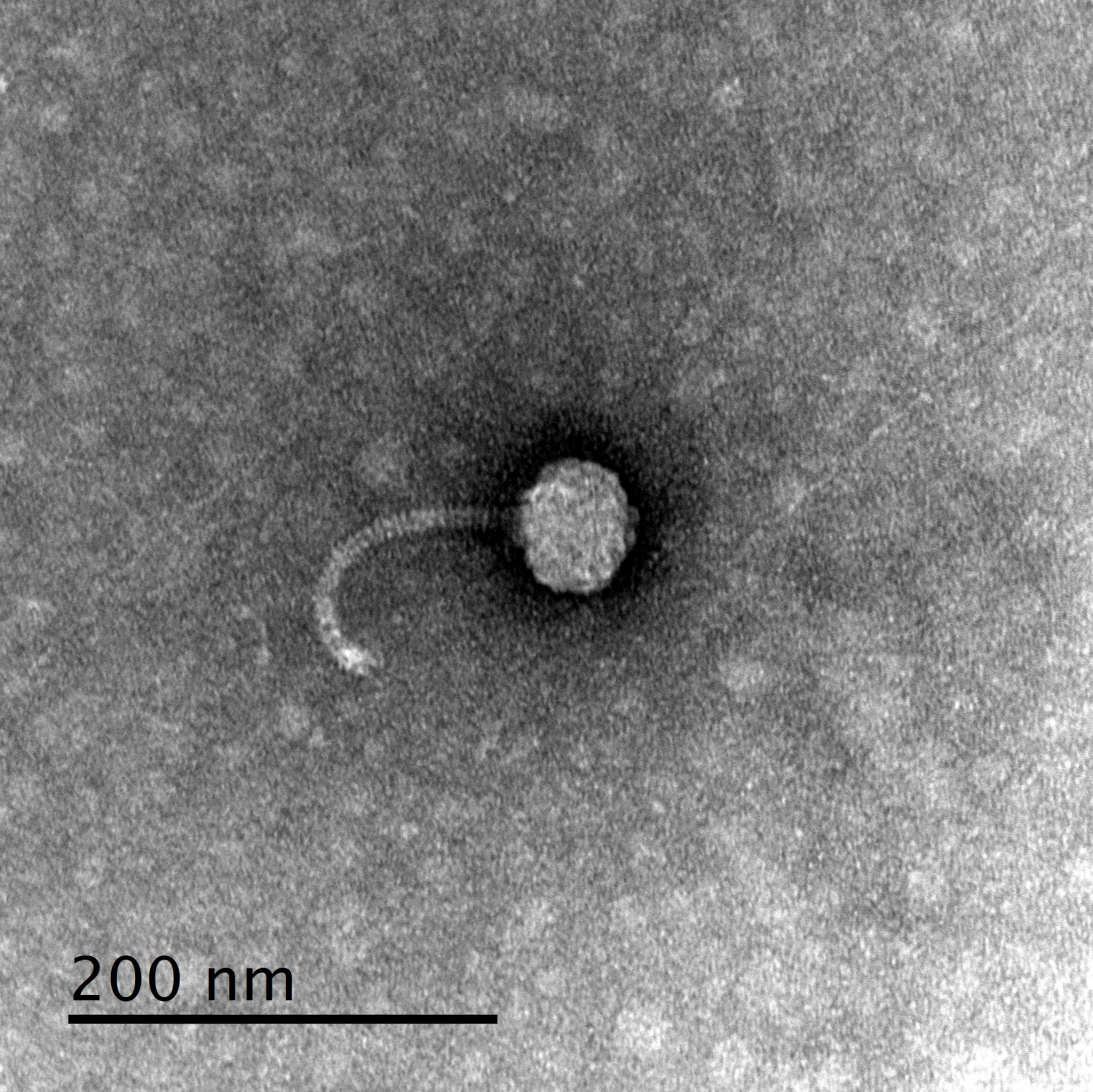
Siphovirus morphology of Statler with a capsid diameter of approximately 65 nm and a tail length of approximately 180 nm (*n* = 1). Image produced by a Zeiss EM900 TEM with an accelerating voltage of 80 kV and 1% uranyl acetate negative staining.

Genomic analysis identified expected genes within the EG cluster: two-tail assembly chaperones (gp35, gp36), a capsid maturation protease (gp24), and an HNH endonuclease (gp54; see Fig. 1). Notably, Statler has a putative cyclic oligonucleotide sequestration protein (gp57; Anti-CBASS 2, Acb2) recently shown to be involved in inhibiting bacterial cyclic-oligonucleotide-based anti-phage signaling system, CBASS (20). Several other EG phages infecting *Microbacterium* also appear to encode Acb2.

## Data Availability

Statler is available at GenBank with accession no. PV915862 and Sequence Read Archive (SRA) SRR31416926. See PhagesDB entry (https://phagesdb.org/phages/Statler/) for collection information, plaque, and phage images.
